# Supplementary material for: Chaperone activation and client binding of a 2-cysteine peroxiredoxin
Source: Nat Commun. 2019 Feb 8;10:659. doi: 10.1038/s41467-019-08565-8 (PMC6368585; doi:10.1038/s41467-019-08565-8)
Supplement: Supplementary file 1 — Supplementary Information [file 41467_2019_8565_MOESM1_ESM.pdf]

# Chaperone Activation and Client Binding of a 2-Cysteine Peroxiredoxin

F. Teixeira, E. Tse et al

## Supplementary Methods

### Sedimentation velocity analytical ultracentrifugation (SV-AUC) measurements

Complexes between $mTXNPx_{red}$ and luciferase were prepared as followed. As before, $mTXNPx_{red}$ was prepared by incubating 10 µM mTXNPx in 40 mM Hepes (pH 7.5), 5 mM DTT at 30°C for 30 min. Then, 1 µM luciferase was added and the complex was formed by slowly heating the sample from 30°C to 42°C (~ 10 min). After the incubation, soluble proteins were separated from insoluble protein by centrifugation. No pellet was detected when in samples that contained both luciferase and $mTXNPx_{red}$. Next, 0.5 mM EDTA and 50 mM NaCl was added. SV-AUC was carried out using 420 µl sample loaded into two-sector Epon centerpieces with 1.2 cm path-length in an An60 Ti rotor in a Beckman Optima XL-I analytical ultracentrifuge, and run at 44 krpm at 5°C. Measurements were completed in intensity mode. Sedimentation of the samples was monitored continuously by absorbance at 280 nm. All SV-AUC data were analyzed using UltraScan 4 software, version and fitting procedures were completed on XSEDE clusters at the Texas Advanced Computing Center (Lonestar, Stampede) through the UltraScan Science Gateway (https://www.xsede.org/web/guest/gateways-listing). The partial specific volume (vbar) of the proteins and the $mTXNPx_{red}$:luciferase complex was estimated within UltraScan III based on the published protein sequences [1]. Raw intensity data were converted to pseudo-absorbance by using the intensity of the air above the meniscus as a reference and edited. Next, 2-dimensional sedimentation spectrum analysis (2DSA) was performed [2] to subtract time-invariant noise and the meniscus was fit using ten points in a 0.05-cm range. Arrays were fit using an S range of 1–15 S, an f/f0 range of 1–4 with 64 grid points for each, 10 uniform grid repetitions and 400 simulation points. 2DSA was then repeated at the determined meniscus to fit radially invariant and time-invariant noise together using ten iterations. The 2DSA solution was validated by Monte-Carlo analysis (2DSA-MC). Furthermore, the data was refined by a genetic algorithm (GA), which uses an evolutionary based approach using random cross-over, mutations and deletion operations to alter the solute characteristics of the 2DSA

solutes to eliminate false positive solutions [3]. This refinement was followed by a validation Monte-Carlo analysis.

**Determination of peroxidase activity**

Determination of tryparedoxin peroxidase activity was performed according to the published literature[4]. Briefly, reaction mixtures were prepared in 50 mM Tris-HCl, pH 8.0, 1 mM EDTA and contained 200 µM NADPH, 0.5 U $ml^{-1}$ trypanothione reductase (TR), 50 µM trypanothione disulfide ($TS_2$), 5 µM tryparedoxin (TXN1) and 1 µM mTXNPx (wild type, Δ5mTXNPx, mTXNPxY33A or mTXNPxR34A). TXN1 and TR were purified according to[4]. Reactions were started by adding 70 µM hydrogen peroxide to the sample, and NADPH consumption was followed at 340 nm. Reactions were performed at 25ºC and monitored with a Shimadzu UV-2401 PC spectrophotometer (Shimadzu Corporation).

**Chaperone activity of two cytosolic TXNPx isoforms of L. infantum**

Cytosolic cTXNPx1 and cTXNPx2 enzymes of *L. infantum* were cloned, expressed and purified from *E. coli*, according to the protocols established for mitochondrial mTXNPx [5]. After removal of the N-terminal histidine tag by thrombin proteolysis, proteins were assayed for chaperone activity using the luciferase thermal aggregation assay (see main manuscript for details).

**Purification of select mTXNPx-Bpa mutant variants and chaperone activity assay**

Purification and reduction of mTXNPx-Y73Bpa and mTXNPx-Y111Bpa were conducted as described for wild-type mTXNPx in the main text. To investigate the chaperone activity of the purified proteins, their influence on the thermal aggregation of firefly luciferase was assessed. Native luciferase (0.1 µM) was incubated either alone or in the presence of a 20-fold molar excess of reduced wild-type mTXNPx or the Bpa mutant variants at 42°C for 10 min in the presence of 0.2 mM DTT. After the incubation, the samples were spun down and the soluble supernatant was loaded onto a 14% SDS-PAGE.

**Differential *in vivo* thiol trapping with NEM**

Cells expressing either wild-type mTXNPx or the Bpa-mutants were cultivated in LB as described in the main text. Afterwards, the *in vivo* redox status of mTXNPx was assessed as previously described[6]. Briefly, cells were treated with trichloroacetic acid (10%) for 30 min on ice. Subsequently, samples were centrifuged (20,000xg 20 min, 4°C) and the precipitated proteins were resuspended in DAB buffer (6 M Urea, 200 mM Tris-HCl pH 8.5, 10 mM EDTA, and 0.5% w/v SDS) +/- 100 mM N-ethylmaleimide (NEM). Upon incubation for 30 min at 25°C, the samples were again precipitated with TCA and separated on SDS-PAGE under reducing or non-reducing conditions and visualized by western blot analysis using a polyclonal anti-mTXNPx antibody.

## Supplementary Table 1. Summary of *in vivo* crosslinking results

| Bpa position | EXP1 | | EXP2 | | EXP3 | | EXP4 | | EXP5 | | Group | Position |
|---|---|---|---|---|---|---|---|---|---|---|---|---|
| | 30°C | 45°C | 30°C | 45°C | 30°C | 45°C | 30°C | 45°C | 30°C | 45°C | | |
| Y30 | ++ | ++ | + | + | | | + | + | | | 2 | surface |
| Y33 | ++ | ++ | + | + | | | | | | | 2 | surface |
| F45 | + | +++ | + | ++ | - | - | | | | +++ | 1 | buried |
| Y63 | - | - | - | - | | | | | | | 3 | surface |
| Y67 | + | +++ | - | + | - | +++ | | | + | +++ | 1 | surface |
| F71 | + | +++ | - | ++ | + | +++ | | | + | +++ | 1 | buried |
| F72 | + | +++ | + | + | | | + | +++ | + | +++ | 1 | buried |
| Y73 | - | ++ | | | | | - | +++ | - | ++ | 1 | buried |
| F77 | + | +++ | - | + | + | +++ | | | + | ++ | 1 | A-type interface |
| F79 | - | + | - | + | + | +++ | + | +++ | + | +++ | 1 | A-type interface |
| F88 | + | +++ | + | ++ | + | +++ | | | + | +++ | 1 | surface |
| F95 | + | +++ | + | + | | | - | ++ | + | ++ | 1 | buried |
| Y111 | + | + | + | + | + | + | + | + | | | 3 | surface |
| Y145 | - | - | + | + | + | + | | | | | 3 | N-terminal |
| F160 | - | + | + | - | - | - | - | - | - | - | 3 | buried |
| F192 | - | - | + | + | - | - | - | - | | | 3 | buried |
| Y194 | | | - | + | - | - | - | + | + | + | 0 | surface |

Degree of Crosslink: **-** no crosslinks or presence of few, highly discrete higher migrating bands; **+** minor smear of higher migrating bands; **++** moderate smear of higher migrating bands; **+++** substantial smear of higher migrating bands.

## Supplementary Table 2 (Related to Table 2). Quantitative Cross-linking (qCL) of $mTXNPx_{red}$ and $mTXNPx_{ox}$ using DSA

| Heavy-CL | Light-CL | CL-Peptides[1] | Log2 (H/L)-average | Favored CL |
|---|---|---|---|---|
| Red (30°C) | Red (30°C) | 1-2 | 0.26 | - |
| Red (30°C) | Red (30°C) | 1-2 | 0.40 | - |
| Red (30°C) | Red (30°C) | 1-2 | 0.37 | - |
| Red (30°C) | Red (30°C) | 1-3 | 0.36 | - |
| Red (30°C) | Red (30°C) | 1-3 | 0.27 | - |
| Red (30°C) | Ox (30°C) | 1-3 | 2.32 | Red (30°C) |
| Red (30°C) | Ox (30°C) | 1-3 | 2.38 | Red (30°C) |
| Red (30°C) | Ox (30°C) | 1-3 | 2.25 | Red (30°C) |
| Red (30°C) | Ox (30°C) | 1-3 | 1.98 | Red (30°C) |
| Red (30°C) | Red (42°C) | 1-3 | 1.40 | Red (30°C) |
| Red (30°C) | Red (42°C) | 1-3 | 1.40 | Red (30°C) |

[1]Pep1: $K_{123}$GGLGEMHIPVLADK; Pep 2: $K_{122}K_{123}$GGLGEMHIPVLADK; Pep 3: $K_{122}$GGLGEM*HIPVLADK; *+16 Da

**Supplementary Table 3. List of oligonucleotides used in this work**

| A.A. Substitution | Primer sequence |
|---|---|
| Y30 | 5' ATGAATCTGGACTAGCAGATGTACCGTA 3' |
| Y33 | 5' CTGGACTATCAGATGTAGCGTACAGCGACTGTC 3' |
| F45 | 5' GAAGCTGCGCCACAGTAGTCCGGCCAGGCTGTC 3' |
| Y63 | 5' GATATAAACATGAACGACTAGAAGGGCAAGTACATTGTGC 3' |
| Y67 | 5' GAACGACTACAAGGGCAAGTAGATTGTGCTGTTTTTCTATC 3' |
| F71 | 5' GCAAGTACATTGTGCTGTAGTTCTATCCGATGGACTTC 3' |
| F72 | 5' CAAGTACATTGTGCTGTTTTAGTATCCGATGGACTTCACC 3' |
| Y73 | 5' GTACATTGTGCTGTTTTTCTAGCCGATGGACTTCACCTTC 3' |
| F77 | 5' GTTTTTCTATCCGATGGACTAGACCTTCGTTTGCCCGACC 3' |
| F79 | 5' CTATCCGATGGACTTCACCTAGGTTTGCCCGACCGAGATC 3' |
| F88 | 5' CCGAGATCATTGCGTAGTCGGATCGCCACG 3' |
| F95 | 5' GATCGCCACGCCGACTAGGAGAAGCTAAACAC 3' |
| Y111 | 5' CGTGCGATTCGGTGTAGTCTCACCTGGCGTG 3' |
| Y145 | 5' GAGATCGCTCGTGACTAGGGTGTGCTGATCGAG 3' |
| F160 | 5' GCTCTTCGAGGGCTCTAGATCATCGACAAGAAG 3' |
| F192 | 5' CGCGTGCTGGAGGCTTAGCAGTACGCGGACGAG 3' |
| Y194 | 5' CTGGAGGCTTTCCAGTAGGCGGACGAGAATGG 3' |
| F221 | 5' CACAAAAGCAGGCGAGTAGTTCGAGAAGAACATGTG 3' |

*Mutations introduced by site directed mutagenesis are underlined.

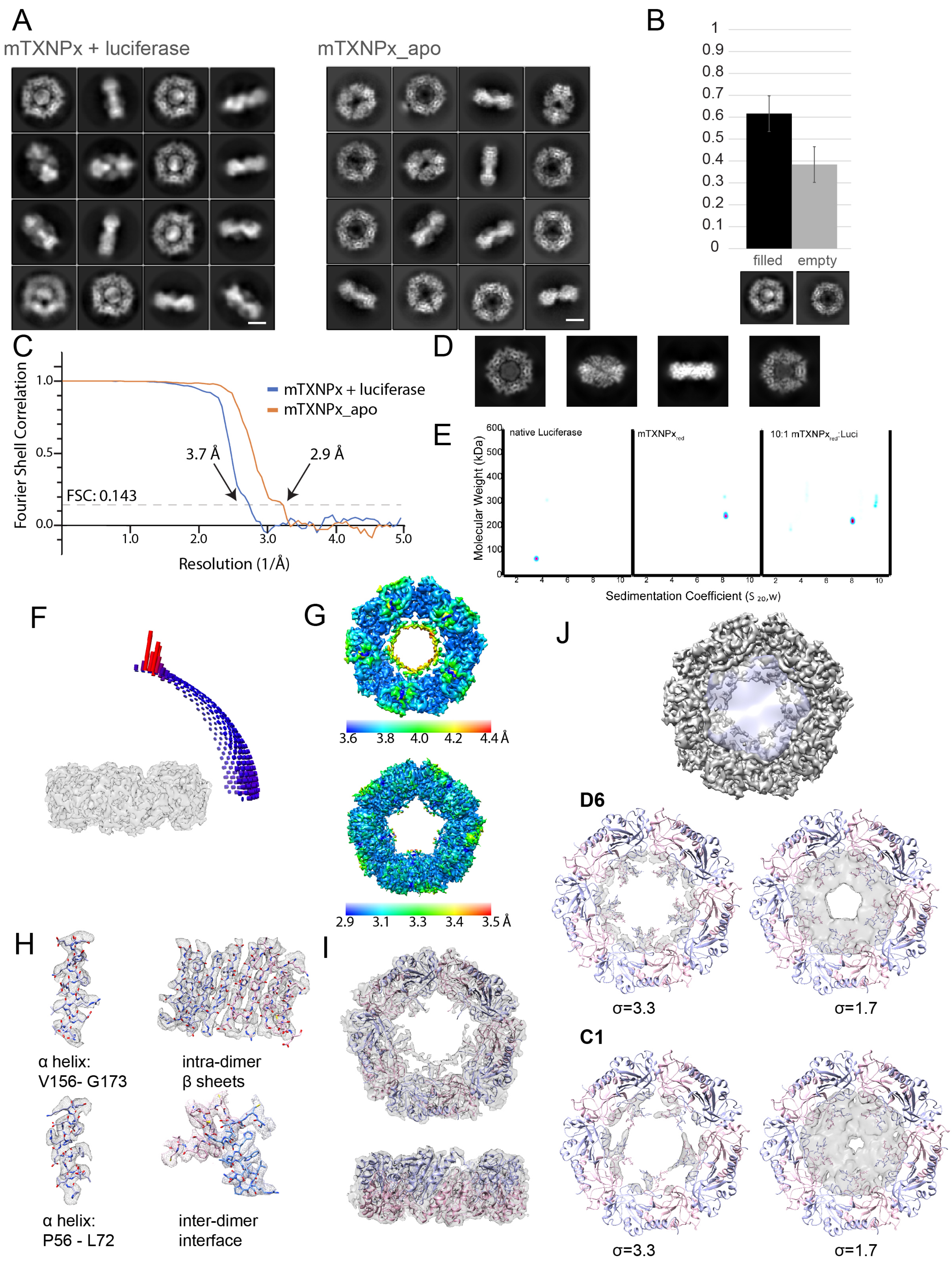

A
mTXNPx + luciferase
mTXNPx_apo
B
1
0.9
0.8
0.7
0.6
0.5
0.4
0.3
0.2
0.1
0
filled
empty
C
Fourier Shell Correlation
1.0
0.5
0.0
FSC: 0.143
3.7 Å
2.9 Å
mTXNPx + luciferase
mTXNPx_apo
1.0
2.0
3.0
4.0
5.0
Resolution (1/Å)
D
E
Molecular Weight (kDa)
native Luciferase
mTXNPx red
10:1 mTXNPx red:Luci
Sedimentation Coefficient (S 20,w)
F
G
3.6
3.8
4.0
4.2
4.4 Å
2.9
3.1
3.3
3.4
3.5 Å
H
α helix:
V156- G173
intra-dimer
β sheets
α helix:
P56 - L72
inter-dimer
interface
I
J
D6
σ=3.3
σ=1.7
C1
σ=3.3
σ=1.7

**Supplementary Figure 1. Cryo-EM data (related to Figure 1)**

**A.** Representative reference-free 2D projection averages of cryo-EM datasets for $mTXNPx_{red}$ incubated with luciferase and without (apo). Scale bar equals 5 nm. **B.** Fraction of the luciferase-bound and free particles from the dataset. Fractions were determined by tallying the number of particles in the top-view oriented classes that contain central density, which thereby corresponds to bound luciferase, to empty rings. Values represent means ± SD from 3 separate datasets. **C**. Gold standard FSC curves of the $mTXNPx_{red}$:luciferase and $mTXNPx_{red}$ 3D reconstructions with imposed D5 symmetry determined at 3.7 and 2.9 Å resolution, respectively by 0.143 FSC cutoff. **D.** Example projection averages of the $mTXNPx_{red}$:luciferase final map. **E** Two-dimension genetic analysis (GA-MC) plot of molecular weight MW *versus* sedimentation coefficient ($S_{20,W}$). Complexes between $mTXNPx_{red}$ and luciferase were formed by slowly heating both proteins together from 30°C to 42°C. About 25% of $mTXNPx_{red}$ molecules sediment at 10 S and an estimated molecular weight of 300 kDa, consistent with one luciferase molecule bound to one $mTXNPx_{red}$ decamer. Native luciferase, which sediments at 3.8S and a MW of 63 kDa, and client-free $mTXNPx_{red,}$ which sediments at 8 S and an estimated molecular weight of 220 kD were analyzed as controls. **F.** Euler angle distribution of the particles used in the $mTXNPx_{red}$:luciferase 3D reconstruction. **G.** Local resolution maps of the $mTXNPx_{red}$:luciferase and $mTXNPx_{red}$ with resolution colored according to the spectrum bars. **H.** High resolution features from the $mTXNPx_{red}$:luciferase map extracted to demonstrate the fit of the refined atomic model in various regions. **I.** 3D reconstruction of $mTXNPx_{red}$:luciferase without imposing symmetry (C1) to demonstrate presence of protruding densities into the $mTXNPx_{red}$ lumen. Top and side views are shown with the docked model of the dodecamer. **J**. Focused refinement of the central density for the $mTXNPx_{red}$:luciferase complex that involved a mask (purple density) around the central density during the 3D refinement (top image). The final maps with the dodecamer model are shown at indicated sigma values for the threshold for refinements performed with D6 and C1 imposed symmetry.

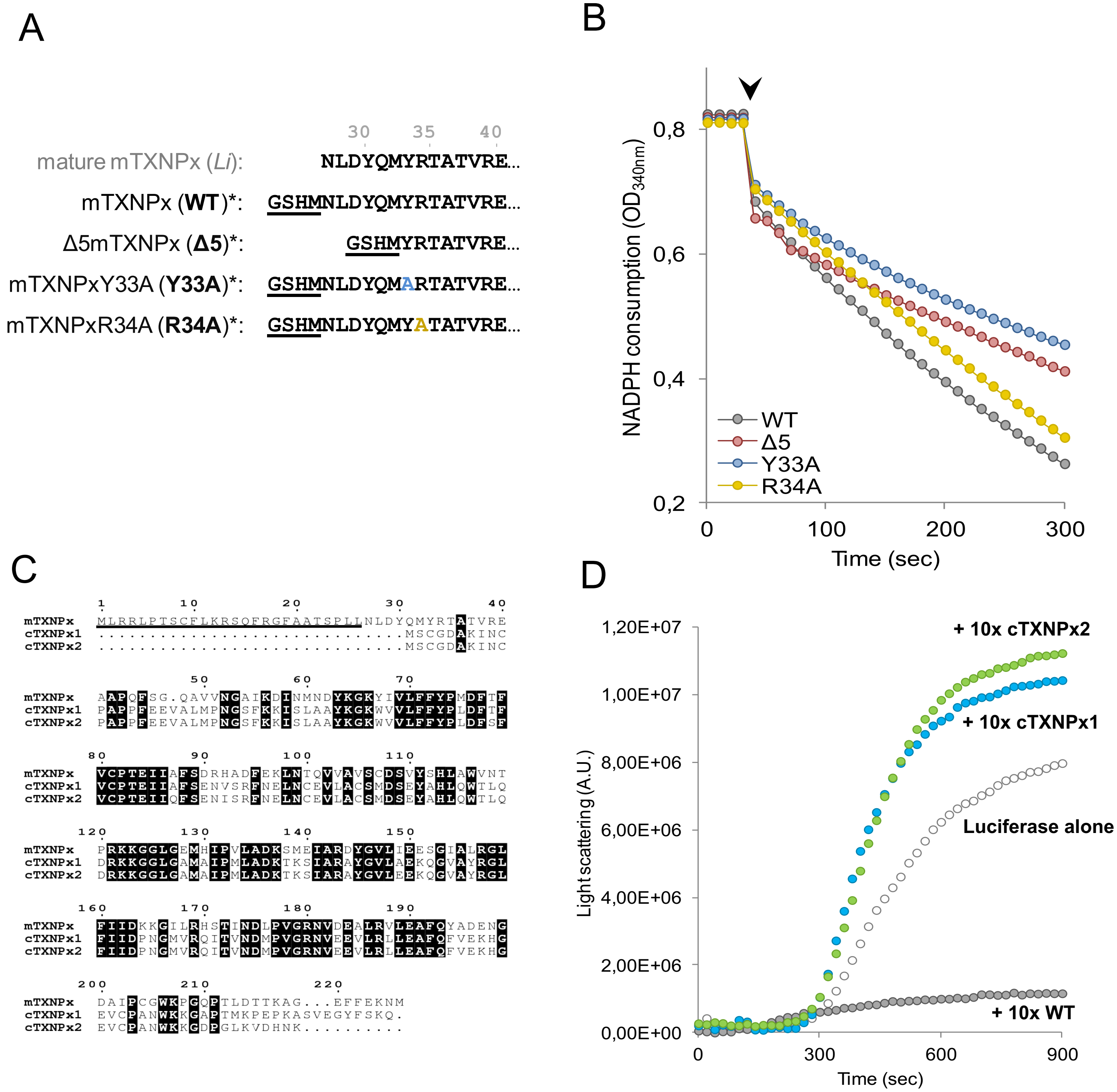
A
30 35 40
mature mTXNPx (Li): NLDYQMYRTATVRE...
mTXNPx (WT)*: GSHMNLDYQMYRTATVRE...
Δ5mTXNPx (Δ5)*: GSHMYRTATVRE...
mTXNPxY33A (Y33A)*: GSHMNLDYQMARTATVRE...
mTXNPxR34A (R34A)*: GSHMNLDYQMYATATVRE...
B
NADPH consumption (OD340nm)
0,8
0,6
0,4
0,2
WT
Δ5
Y33A
R34A
0
100
200
300
Time (sec)
C
mTXNPx
cTXNPx1
cTXNPx2
D
Light scattering (A.U.)
1,20E+07
1,00E+07
8,00E+06
6,00E+06
4,00E+06
2,00E+06
0,00E+00
+ 10x cTXNPx2
+ 10x cTXNPx1
Luciferase alone
+ 10x WT
0
300
600
900
Time (sec)

**Supplementary Figure 2. Peroxidase Activity of mTXNPx mutant variants and *in vitro* chaperone activity of cytosolic TXNPx homologues in Leishmania**

**A.** Alignment of the N-terminal amino acid sequences of mature mTXNPx (i.e., mTXNPx without the mitochondrial targeting sequence) as well as of the purified mTXNPx variants. All purified mTXNPx proteins contain a three-aa-scar sequence, i.e., GSH, which is a remnant of the thrombin cleavage. **B.** Tryparedoxin peroxidase enzymatic activity. Reaction mixtures contained 200 µM NADPH, 0.5 U $ml^{-1}$ trypanothione reductase (TR), 50 µM trypanothione disulfide ($TS_2$), 5 µM tryparedoxin 1 and 1 µM mTXNPx (wild type, Δ5mTXNPx, mTXNPxY33A or mTXNPxR34A). Reactions were started by addition of 70 µM $H_2O_2$ at 30 sec (indicated by arrowhead) and peroxidase activity was followed by monitoring NADPH consumption at 340 nm. **C.** Sequence alignment of the predicted amino acid sequence of mature mTXNPx and of two cytosolic peroxiredoxins of *L. infantum* (cTXNPx1 and cTXNPx2). Strict identity across all sequences is shown in reversed typing. The mitochondrial targeting sequence of mTXNPx, which is absent in the mature protein is underlined. **D.** Influence of mTXNPx, cTXNPx1 and cTXNPx2 on the thermal aggregation of luciferase. Native luciferase (0.1 µM) was incubated in the absence or presence of a 10-fold molar excess of TXNPx at 42°C, and light scattering (expressed in arbitrary units, A.U.) was monitored at 360 nm. Reactions were carried out under reducing conditions (0.2 mM DTT).

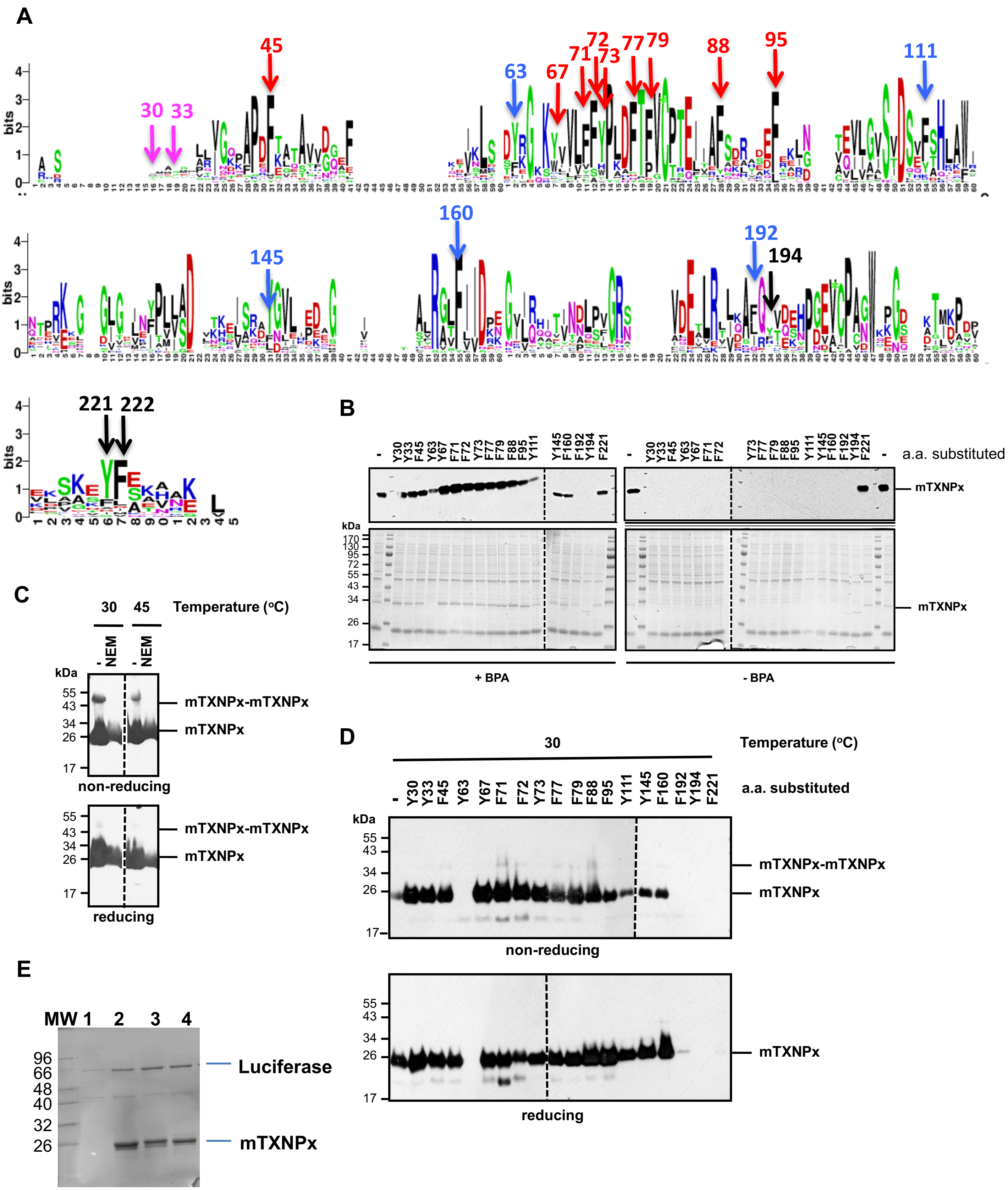
A
45
30 33
63
67
71
72
73
77
79
88
95
111
145
160
192
194
221 222
bits
B
a.a. substituted
Y30 Y33 F45 Y63 Y67 F71 F72 Y73 F77 F79 F88 F95 Y111 Y145 F160 F192 Y194 F221
mTXNPx
kDa
170 130 95 72 55 43 34 26 17
+ BPA
- BPA
C
Temperature (°C)
30 45
- NEM
kDa
mTXNPx-mTXNPx
mTXNPx
non-reducing
reducing
D
30
Temperature (°C)
a.a. substituted
mTXNPx-mTXNPx
mTXNPx
non-reducing
reducing
E
MW 1 2 3 4
96 66 48 40 32 26
Luciferase
mTXNPx

**Supplementary Figure 3. Sequence logo of mTXNPx and mTXNPx-Bpa mutant characterization (related to Figure 2)**

**A.** Sequence logo of mTXNPx. All residues that were substituted for Bpa are indicated in color. The color code correspond to the cross-linking results shown in Fig. 2 and Table S1. with Group 1 residues shown in red, Group 2 residues shown in magenta and Group 3 residues shown in blue. mTXNPx variants with codon replacements at position 194, 221, 222 were constructed but were not further studied (see text for details). **B**. Expression of select mTXNPx WT and Bpa-mutants in the presence (left panel) or absence (right panel) of Bpa in the growth media. Samples were loaded onto SDS-PAGE (lower panel) and western blot analysis was conducted using anti-mTXNPx antibodies (upper panel). **C/D**. *In vivo* redox status of His-mTXNPx WT and Bpa-containing mutants. Cells expressing either His-tagged mTXNPx (**C**) or Bpa-containing mutants (**D**) were grown as described. To trap the *in vivo* redox state of the proteins, cells were acidified with TCA. Pellets were resuspended in denaturing buffer containing the thiol-alkylating reagent NEM. After irreversibly alkylating all reduced thiols, the samples were loaded onto non-reducing (top panel) or reducing (bottom panel) SDS-PAGE and mTXNPx was visualized by westernblot. **E.** Influence of a 20-fold excess of wild-type and Bpa-mutant variants on the aggregation of thermally unfolding luciferase. Luciferase (0.1 μM) was incubated either alone (lane 1) or in the presence of a 20-fold molar excess of reduced wild-type mTXNPx (lane 2), Y73Bpa (lane 3) or Y111Bpa (lane 4) at 42°C for 10 min. After the incubation, the samples were spun down and the soluble supernatant was loaded onto a 14% SDS-PAGE. While most of the luciferase aggregates and disappears from the soluble supernatant, presence of wild-type and mutant mTXNPx maintains the solubility of the enzyme.

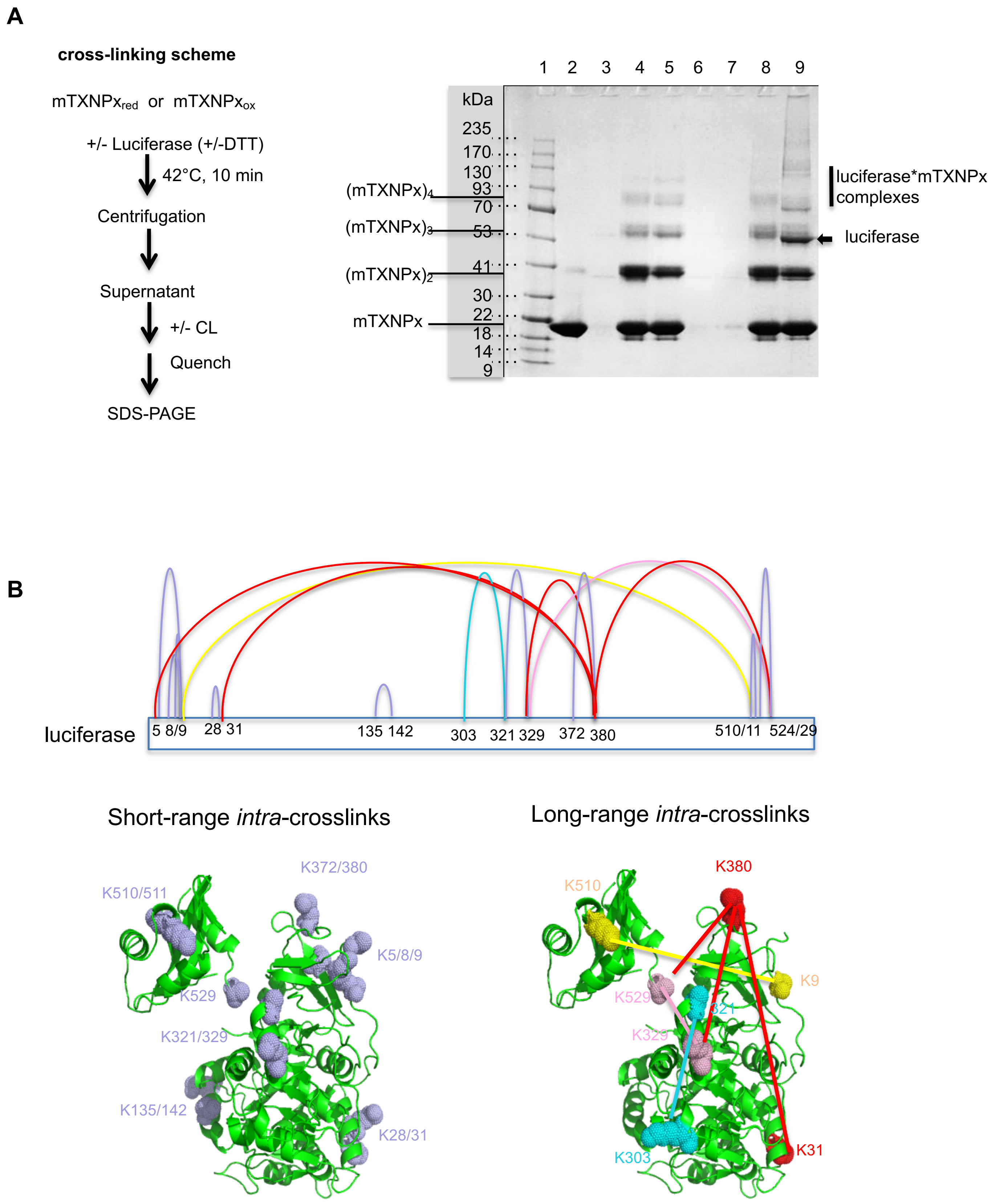
A
cross-linking scheme
mTXNPxred or mTXNPxox
+/- Luciferase (+/-DTT)
42°C, 10 min
Centrifugation
Supernatant
+/- CL
Quench
SDS-PAGE
1 2 3 4 5 6 7 8 9
kDa
235
170
130
93
70
53
41
30
22
18
14
9
(mTXNPx)4
(mTXNPx)3
(mTXNPx)2
mTXNPx
luciferase*mTXNPx complexes
luciferase
B
luciferase
5 8/9
28 31
135 142
303
321 329
372 380
510/11 524/29
Short-range intra-crosslinks
Long-range intra-crosslinks
K510/511
K372/380
K5/8/9
K529
K321/329
K135/142
K28/31
K510
K380
K9
K529
321
K329
K303
K31

**Supplementary Figure 4. *In vitro* cross-linking scheme and visualization of cross-linkage results (related to Figure 3). A**. Chaperone-active $mTXNPx_{red}$ or chaperone-inactive $mTXNPx_{ox}$ (5 µM) were incubated in the absence of presence of 0.5 µM luciferase at 42°C for 10 min. After centrifugation, the soluble supernatants were collected and cross-linked for 15 min using the amine-specific cross-linker DSS or DSA. Soluble supernatants were loaded onto reducing SDS-PAGE. An SDS-PAGE in which DSS was used as the cross-linking reagent is shown. SDS-PAGE of proteins cross-linked with DSS was similar in appearance (not shown). Lanes 1: Marker; 2: $mTXNPx_{ox}$; 3: luciferase; 4: $mTXNPx_{ox}$ + CL; 5: $mTXNPx_{red}$ + CL; 6: luciferase + CL; 7: luciferase + DTT + CL; 8: $mTXNPx_{ox}$ + luciferase + CL; 9: $mTXNPx_{red}$ + luciferase + CL. No soluble luciferase was observed except in lane 9 due to its almost complete aggregation in the absence of functional chaperones. Additional preparations of $mTXNPx_{red}$ + luciferase + CL (DSS or DSA) were made and separated by SDS-PAGE (not shown) for in-gel trypsin digest and MS/MS analysis. Gel pieces were excised from 70-93 kDa or 93 kDa-well. **B**. Crosslinking pattern of thermally unfolded luciferase in complex with $mTXNPx_{red}$. $mTXNPx_{red}$ – luciferase complexes were formed, crosslinked with DSS and excised (Supplementary Figure 3A, lane 9). After tryptic digestion, the peptides were analyzed by MS/MS analysis. All crosslinks were *intra*-crosslinks within the luciferase monomer. Short-range (purple) and long range (yellow, red, cyan) cross-links are indicated on the linear structure (upper panel) or on the crystal structure of firefly luciferase (pdb 1LCI). Results with both cross-linkers are shown (Supplementary Data 1).

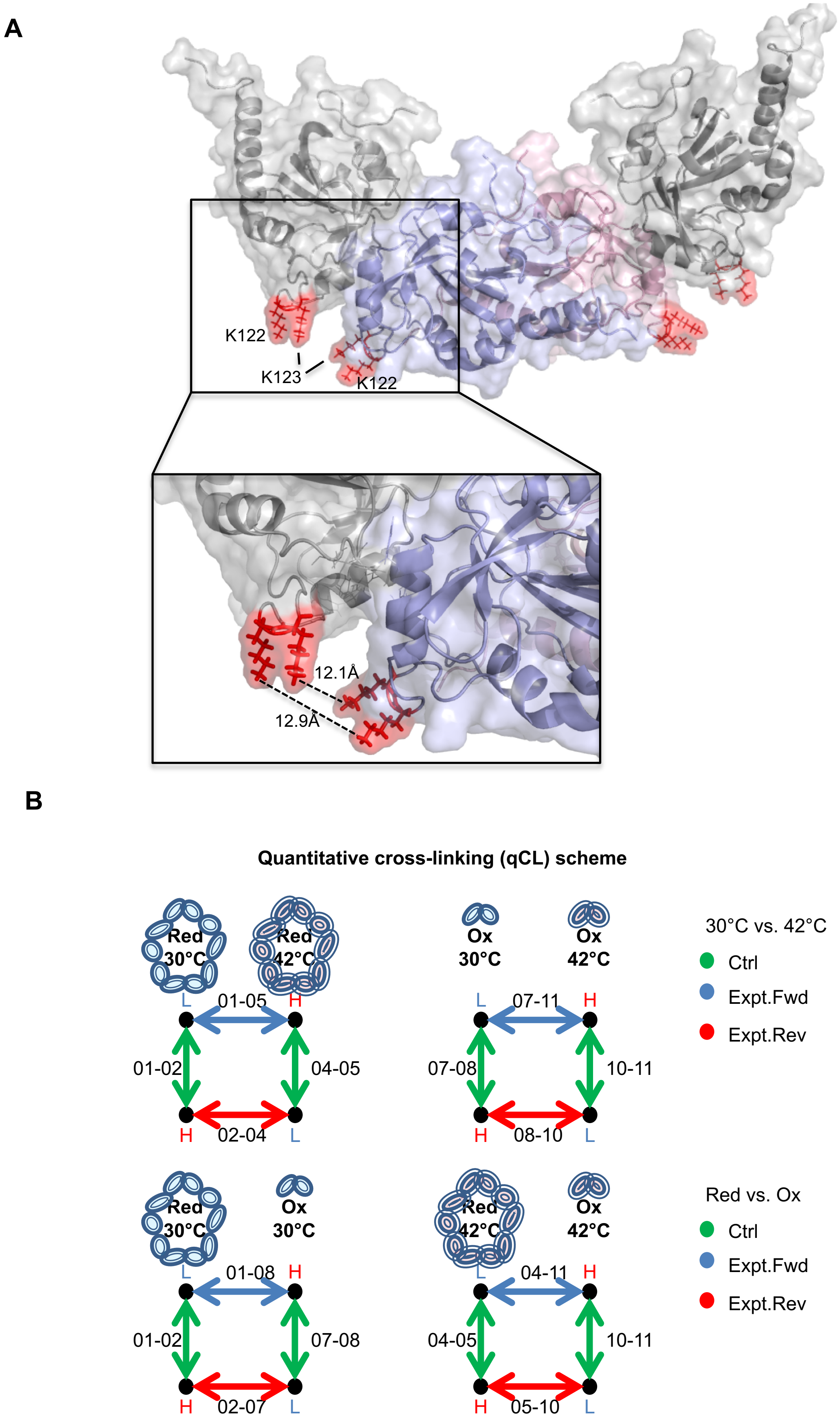
A
K122
K123
K122
12.1Å
12.9Å
B
Quantitative cross-linking (qCL) scheme
Red 30°C
Red 42°C
Ox 30°C
Ox 42°C
L
H
01-05
01-02
04-05
02-04
07-11
07-08
10-11
08-10
01-08
07-08
02-07
04-11
04-05
05-10
30°C vs. 42°C
Ctrl
Expt.Fwd
Expt.Rev
Red vs. Ox
Ctrl
Expt.Fwd
Expt.Rev

**Supplementary Figure 5. Quantitative cross-linking (qCL) of heat-activated mTXNPx (related to Figure 5). A.** Location of K122/K123 is indicated in two adjacent dimers of the decamer. The distance between the lysine residues in the neighboring subunits ranges between 12.1 and 12.9 Å. No significant cross-links are expected once mTXNPx dissociates into the dimers. **B**. Quantitative cross-linking (qCL) experiments were performed in an identical fashion with respect to the reduction, temperature incubation, centrifugation, and cross-linking except that no luciferase was added and that cross-linking was performed using either isotopically-light or isotopically-heavy forms of the cross-linking reagent separately for each condition. For both cross-linking reagents used (DSA and DSS), four distinct sample conditions ($mTXNPx_{ox}$ at 30˚C, $mTXNPx_{ox}$ at 42˚C, $mTXNPx_{red}$ at 30˚C, or $mTXNPx_{red}$ at 42˚C) were prepared, split into 2 equal volumes, and cross-linked using a 0.1 mM final concentration of the isotopically-light and isotopically-heavy forms of the cross-linking reagent. These 8 preparations were then combined in equal amounts. Combined samples were subsequently digested in-solution with trypsin and prepared for LC-MS/MS analysis.

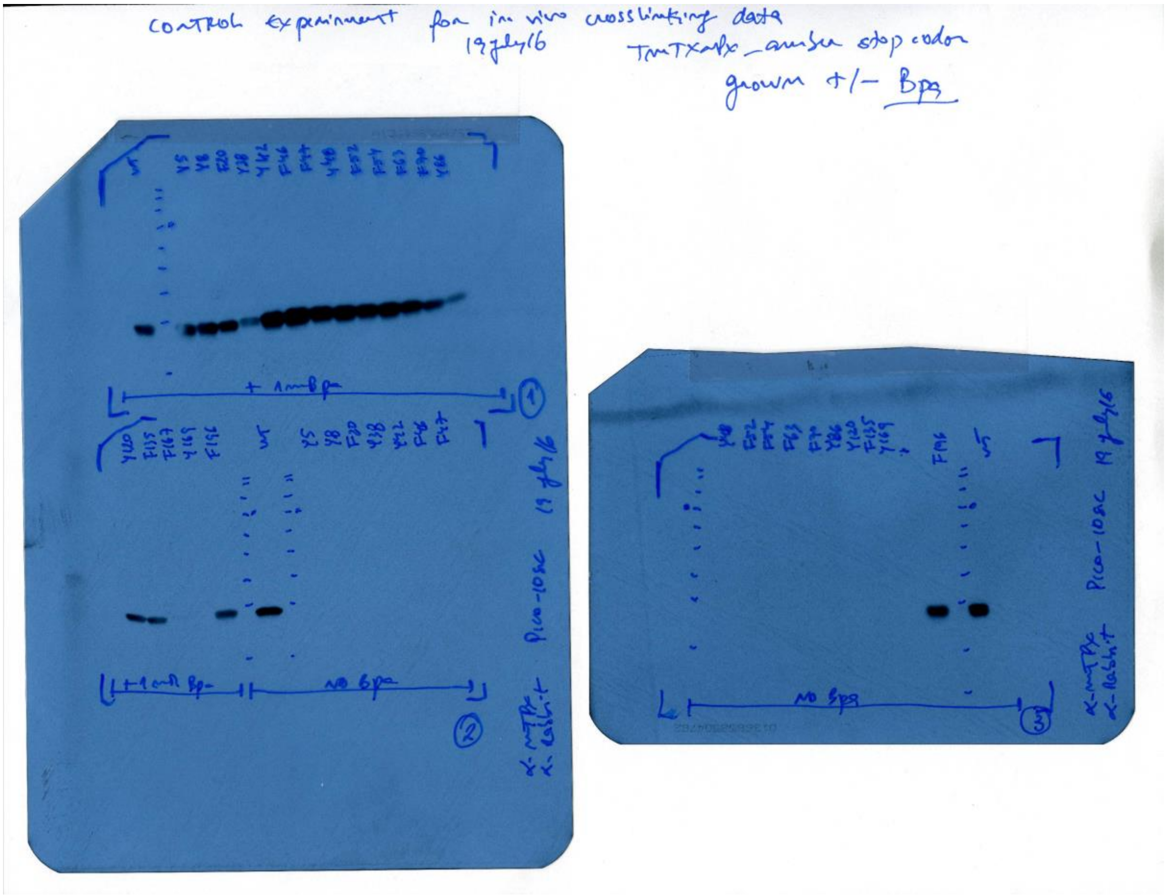


**Supplementary Figure 6. Uncropped Westernblot for Supplementary Figure S3B.**

**Supplementary References**


1. Demeler, B., Brookes, E. & Nagel-Steger, L. Analysis of heterogeneity in molecular weight and shape by analytical ultracentrifugation using parallel distributed computing. *Methods Enzymol* **454**, 87-113 (2009).
2. Brookes, E., Cao, W. & Demeler, B. A two-dimensional spectrum analysis for sedimentation velocity experiments of mixtures with heterogeneity in molecular weight and shape. *Eur Biophys J* **39**, 405-14 (2010).
3. Brookes, E.a.D., B. Genetic Algorithm Optimization for obtaining accurate Molecular Weight Distributions from Sedimentation Velocity Experiments. in *Progress in Colloid and Polymer Science*, Vol. 131 (ed. Wandrey, C.a.C., H.) 78-82 (Springer, 2006).
4. Nogoceke, E., Gommel, D.U., Kiess, M., Kalisz, H.M. & Flohe, L. A unique cascade of oxidoreductases catalyses trypanothione-mediated peroxide metabolism in Crithidia fasciculata. *Biol Chem* **378**, 827-36 (1997).
5. Castro, H. et al. Leishmania mitochondrial peroxiredoxin plays a crucial peroxidase-unrelated role during infection: insight into its novel chaperone activity. *PLoS Pathog* **7**, e1002325 (2011).
6. Teixeira, F. et al. Mitochondrial peroxiredoxin functions as crucial chaperone reservoir in Leishmania infantum. *Proc Natl Acad Sci U S A* **112**, E616-24 (2015).
