# Supplementary material for: Chaperone activation and client binding of a 2-cysteine peroxiredoxin
Source: Nat Commun. 2019 Feb 8;10:659. doi: 10.1038/s41467-019-08565-8 (PMC6368585; doi:10.1038/s41467-019-08565-8)
Supplement: Supplementary file 3 — Description of Additional Supplementary Files [file 41467_2019_8565_MOESM3_ESM.pdf]

Description of Additional Supplementary Files

File Name: Supplementary Data 1
Description: Excel workbook containing all PSMs for mTXNPxred + luciferase at 42°C CLMS experiments and input used to generate the xiNET diagram (Related to Figure 5, Supplementary Figure 4B).

File Name: Supplementary Data 2
Description: Excel workbook containing all PSMs for qCL experiments (related to Table 2, Supplementary Table 2).
